# Ethanol Pharmacokinetics and Alcohol-Related Effects after One-Anastomosis Gastric Bypass: A Prospective before-after Study

**DOI:** 10.1159/ofa/adzag007

**Published:** 2026-09-09

**Authors:** Elchanan Quint, Daniel Porat, Almog Eliyahu Dahan, Ilia Pinsk, Artur Windman, Ivan Kukeev, Arik Dahan, Oleg Dukhno

**Affiliations:** a Department of General Surgery B, Soroka University Medical Center, Beer-Sheva, Israel; b Department of Clinical Pharmacology, School of Pharmacy, Faculty of Health Sciences, Ben-Gurion University of the Negev, Beer-Sheva, Israel; c Department of Clinical Pharmacy, Clalit Health Services, Tel-Aviv, Israel; d Bariatric Surgery Unit, Department of General Surgery B, Soroka University Medical Center, Beer-Sheva, Israel

**Keywords:** Alcohol consumption, Ethanol absorption, Pharmacokinetics, Safety, Weight loss surgery

## Abstract

**Introduction:**

Metabolic-bariatric surgery is associated with altered ethanol pharmacokinetics and increased alcohol-related risk. While these changes have been described after Roux-en-Y gastric bypass and sleeve gastrectomy, data regarding one-anastomosis gastric bypass (OAGB) have not been reported. This exploratory study evaluated changes in alcohol absorption, elimination, and subjective intoxication following OAGB.

**Methods:**

In this prospective before-after interventional study, adults undergoing OAGB were assessed preoperatively and at 1 month and 4 months postoperatively. At each visit, participants ingested 200 mL of red wine (13.5% alcohol by volume; ∼21.3 g ethanol). Breath alcohol concentration (BrAC) was measured at fixed time points over 2 h using a calibrated breathalyzer. Pharmacokinetic parameters, including maximum concentration (C_max_), time to maximum concentration (T_max_), area under the concentration-time curve (AUC_0-inf_), and elimination half-life (t_1/2_), were determined. Subjective alcohol effects were assessed using a standardized symptom questionnaire, and pharmacokinetic-pharmacodynamic relationships were examined.

**Results:**

Ten participants (mean age 42 ± 12.8 years; 60% female) were included in this study. Mean C_max_ increased from 0.43 ± 0.33 g·L^−1^ preoperatively to 1.19 ± 0.30 g·L^−1^ at 1 month and 1.24 ± 0.36 g·L^−1^ at 4 months postoperatively. AUC_0-inf_ increased from 21.0 ± 15.2 to 71.2 ± 31.5 and 91.2 ± 39.3 g·min·L^-1^, respectively. Ethanol elimination half-life was prolonged after surgery, and differences remained statistically significant after BMI standardization. Intoxication symptom scores increased postoperatively and positively correlated with pharmacokinetic parameters, with C_max_ having the strongest association.

**Conclusion:**

OAGB alters ethanol pharmacokinetics, leading to higher peak alcohol concentrations, increased systemic exposure, and enhanced subjective alcohol effects. Even modest alcohol intake may potentially lead to high BrAC levels after OAGB, reflecting the need for clinician-led patient education and advice on safer alcohol use after surgery. Given the small sample size, participant attrition, and short postoperative observation period, the study findings should be interpreted with caution.

## Introduction

Obesity is a chronic condition with a rapidly increasing global prevalence, most notably in high-income populations, where the prevalence of severe obesity is projected to rise from 10% to 20% between 2020 and 2035, posing a substantial challenge to healthcare systems [1]. Medical and behavioral weight loss therapies often fall short for many individuals with obesity, leading to an increased reliance on surgical interventions, i.e., metabolic-bariatric surgery (MBS), which provide significant weight reduction and meaningful improvements in obesity-related comorbidities [2]. Long-term studies show that patients who undergo MBS have lower mortality rates than individuals with comparable clinical profiles who do not undergo surgical intervention [2, 3]. Beyond weight loss, the surgery also leads to improved postoperative quality of life [4].

Despite its well-documented benefits, MBS is associated with certain risks. In addition to potential complications such as bleeding, anastomotic leaks, strictures, ulcers, gallstones, and micronutrient deficiencies, concerns have also been raised regarding postoperative morbidity and mortality unrelated to surgical complications [5]. Robust empirical evidence indicates that individuals undergoing MBS face a higher risk of alcohol-related complications, ranging from increased alcohol consumption to the development of alcohol use disorder (AUD) [6, 7]. Potential mechanisms underlying these alcohol-related complications include altered pharmacokinetics (PK), changes in gut hormones and microbiota, nutritional factors, and alterations in reinforcement and reward pathways, all of which may increase alcohol sensitivity and misuse risk [8]. Several large observational studies have further demonstrated increased rates of alcohol-related morbidity and mortality following MBS. These findings highlight the need to better understand how MBS affects alcohol metabolism and alcohol-related risks [9–12].

As previously noted, one contributing factor to alcohol-related complications after MBS is the alteration in ethanol pharmacokinetics. This phenomenon has been investigated across different metabolic-bariatric procedures with mixed results. Most studies of Roux-en-Y gastric bypass (RYGB) have shown faster alcohol absorption, higher peak blood alcohol concentrations (C_max_), and a prolonged duration of alcohol effect [13–17]. Meanwhile, multiple studies regarding sleeve gastrectomy (SG) and its impact on alcohol PK suggest a more modest effect [18–21] (Table 1).

**Table 1.** Published human studies on the effects of MBS on ethanol PK, categorized by procedure type, time since surgery, and impact on ethanol C_max_

<1 year after MBS				≥1 year after MBS			
RYGB		SG		RYGB		SG	
markedly higher BAC C_max_	modest change in BAC C_max_	markedly higher BAC C_max_	modest change in BAC C_max_	markedly higher BAC C_max_	modest change in BAC C_max_	markedly higher BAC C_max_	modest change in BAC C_max_
[13]		[17]	[19]	[14]	[21]	[22]	[19]
[17]		[18]	[20]	[15]	[23]		[20]
				[16]	[24]		[21]

MBS, metabolic-bariatric surgery; PK, pharmacokinetics; RYGB, Roux-en-Y gastric bypass; SG, sleeve gastrectomy; BAC, blood alcohol concentration; C_max_, maximal concentration.

The newer metabolic-bariatric surgical procedure, one-anastomosis gastric bypass (OAGB), was developed in the late 1990s. This technique is relatively simple, with a short surgical duration and low incidence of adverse events [25]. OAGB is increasingly recognized as an effective and safe metabolic-bariatric procedure and is endorsed by major international professional societies [26, 27]. Since 2016, OAGB has consistently been ranked as the third most common metabolic-bariatric procedure worldwide, following SG and RYGB [28], and in Israel, it is the most prevalent metabolic-bariatric surgical approach [29]. It has been shown to improve obesity-related comorbidities, achieve substantial excess weight loss (EWL%), and enhance postoperative quality of life, while maintaining a relatively low complication rate (7–13%). In addition, OAGB allows for a range of revisional metabolic-bariatric options if needed [30]. However, data on alcohol-related clinical outcomes following OAGB are limited, and its impact on alcohol PK has not yet been systematically examined. This study aims to investigate the absorption and elimination kinetics of alcohol, as well as its subjective effects, in patients who have undergone OAGB.

## Materials and Methods

### Methods

The present exploratory study was a prospective, single-arm, before-after interventional study involving patients undergoing laparoscopic OAGB, all of whom underwent a standardized procedure with a uniform 200-cm biliopancreatic limb in accordance with institutional protocol. Ethanol levels were assessed preoperatively and at 1 month and 4 months postoperatively using breath alcohol concentration (BrAC), a validated, nonintrusive surrogate of blood alcohol concentration (BAC). Measurements were obtained at fixed time points over a 2-h period following ingestion of 200 mL (6.7 oz) of red wine containing 13.5% alcohol by volume, corresponding to approximately 21.3 g of ethanol. Participants fasted for 2 h before alcohol ingestion and were instructed to consume the entire 200-mL glass of wine within 5 min. Measurements were then obtained according to the predefined sampling schedule. An AlcoHAWK PT500 breathalyzer (Quest Products, Pleasant Prairie, WI), cleared by the FDA under 510(k) K080848, was used to measure BrAC. The device was calibrated according to the manufacturer’s instructions.

It has been previously indicated that BrAC, measured by a breathalyzer, closely approximates arterial BAC [31]. BrAC measurements were collected to create individual PK profiles. PK parameters, including AUC_0-inf_, C_max_, T_max_, and t_1/2_, were calculated individually for each participant using PKSolver 2.0 (Excel VBA add-in; Zhang et al., China Pharmaceutical University, Nanjing, China). Group results are presented as mean (SD).

Participants also completed an alcohol-intoxication symptoms questionnaire 60 min after wine ingestion. They were assessed for symptoms including flushing, euphoria, somnolence, dizziness, hyperhidrosis, headache, paresthesia, disorientation, nausea, heat sensation, and diplopia. Each symptom was rated on a 5-point Likert scale (1 = “not at all” to 5 = “very much”). Individual item scores were then summed to calculate a total symptom score for each participant. The relationship between PK parameters and pharmacodynamic (PD) questionnaire scores was evaluated by plotting each subject’s AUC_0-inf_, C_max_, and concentration at 60 min (C_60 min_) against their total symptom score, and computing the linear regression correlation coefficients.

### Inclusion and Exclusion Criteria

The study included adults aged 18–65 years who were scheduled to undergo OAGB at our medical center and had provided written informed consent to participate. Participants had not undergone prior metabolic-bariatric procedures, with the exception of patients with an adjustable gastric band that had been completely opened before the assessment. All participants met the MBS eligibility criteria at the time of study initiation (BMI ≥40 kg/m^2^, or BMI ≥35 kg/m^2^ with obesity-related comorbidities) and were reviewed by the institutional bariatric committee in accordance with the Ministry of Health guidelines. Participants were screened for chronic alcohol use or dependence using the Michigan Alcohol Screening Test (MAST) questionnaire as part of the study evaluation. Exclusion criteria included a BMI <35 kg/m^2^, any previous gastric surgery or MBS other than an adjustable gastric band, and current or past AUD.

### Ethics Approval

The study was approved by the Institutional Review Board (Helsinki Committee) of Soroka University Medical Center, Approval #SOR-20-0324. Written informed consent was obtained from all participants.

### Statistics

Data management and statistical analyses were performed using Microsoft Excel 2013. Categorical variables were presented as frequencies and percentages, and differences were evaluated using the chi-square test. Continuous variables were expressed as mean ± standard deviation (SD). Statistical significance was assessed using a two-tailed paired *t* test to determine whether there were any significant differences between the paired observations; *p* value <0.05 was considered statistically significant.

Sample size considerations were guided by the expectation of a substantial increase in peak alcohol concentrations following surgery, based on findings reported after other gastric bypass procedures. Yet, as this was the first prospective study evaluating ethanol PK following OAGB, a formal a priori power calculation was not performed.

## Results

### Patients

The study included 10 participants with a mean (SD) age of 42 (12.8). Six participants (60%) were female; two participants (20%) had diabetes mellitus, three (30%) had fatty liver disease, and two (20%) had previously undergone an adjustable gastric band procedure. The mean BMI was 41.7 (4.7) kg/m^2^ preoperatively, 38.0 (4.8) kg/m^2^ at 1 month postoperatively, and 33.1 (1.9) kg/m^2^ at 4 months postoperatively (Table 2).

**Table 2.** Patient characteristics

Variables	Group	​
Age, mean (SD)	​	42.0 (12.8)
Female, %		60
Diabetes mellitus, %		20
Fatty liver, %		30
History of adjustable gastric band, %		20
BMI, mean (SD)	Preoperative	41.7 (4.7)
	1-mo postoperative	38.0 (4.8)
	4-mo postoperative	33.1 (1.9)

BMI, body mass index, in kg/m^2^.

### Ethanol Concentrations

Mean ethanol concentrations were higher at all time points 1 month after surgery compared with pre-OAGB values and were further elevated 4 months postoperatively. The maximal concentrations (T_max_) were observed at 15 min in all three visits (Fig. 1). Mean (SD) C_max_ was significantly higher at both 1 month (N = 8) and 4 months (N = 7) after OAGB, reaching 1.19 (0.30) g·L^−1^ and 1.24 (0.36) g·L^−1^, respectively, compared with a presurgery C_max_ of 0.43 (0.33) g·L^−1^ (Table 3). Paired analysis of participants with both pre- and post-surgery measurements demonstrated a mean increase of 0.80 g·L^−1^ (95% CI: 0.46–1.14 g·L^−1^, *p* < 0.001) and 0.83 g·L^-1^ (95% CI: 0.28–1.37 g·L^−1^, *p* < 0.05), respectively. AUC_0-inf_ was also significantly increased, with values of 71.2 (31.5) g·min·L^−1^ at 1 month and 91.2 (39.3) g·min·L^−1^ at 4 months, compared with 21.0 (15.2) g·min·L^-1^ presurgery. Paired analysis demonstrated a mean increase of 52.6 g·min·L^−1^ (95% CI: 29.3–75.9 g·min·L^−1^, *p* < 0.01) and 74.7 g·min·L^−1^ (95% CI: 43.4–106.1 g·L^−1^, *p* < 0.01), respectively. Following OAGB, elimination t_1/2_ was prolonged, measuring 34.1 (13.9) min at 1 month and 43.4 (16.8) min at 4 months, compared with 20.7 (15.0) min preoperatively; paired analysis: 11.7 min (95% CI: −15.6–39.0 min, *p* = 0.28) and 30.3 min (95% CI: 17.9–42.6 g·L^−1^, *p* < 0.01), respectively (Fig. 1) (Table 3). Given the substantial postoperative weight loss, PK results were additionally normalized to each participant’s BMI at the corresponding study visit. Despite this adjustment, the observed differences remained statistically significant (Fig. 2) (Table 4). Individual PK profiles are shown in Figure 3, demonstrating consistently higher ethanol levels after OAGB.

**Fig. 1.**
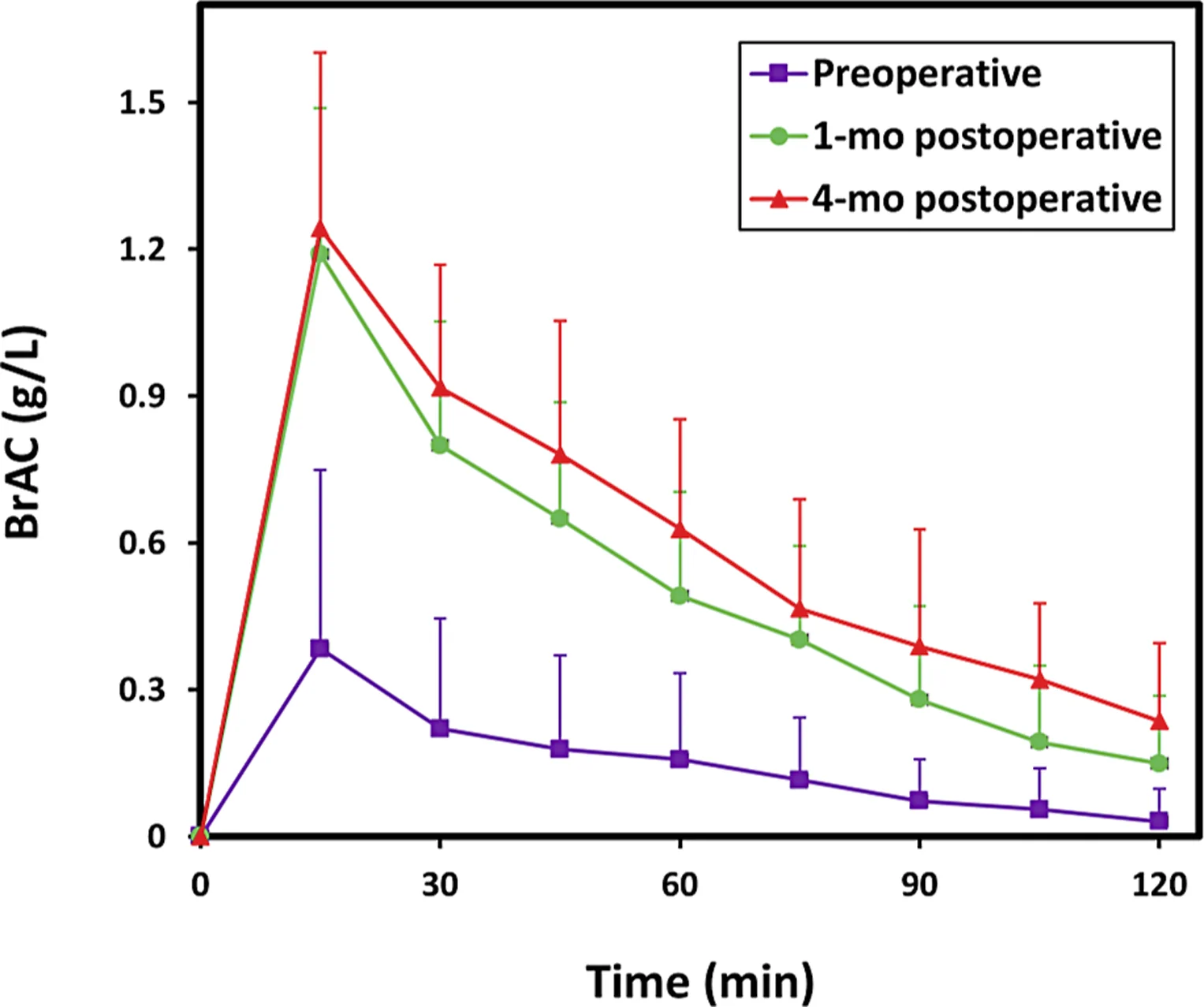
BrAC-time profiles before surgery (purple squares) and at 1 month (green circles) and 4 months (red triangles) after OAGB, following wine ingestion. Bars represent standard deviation. BrAC, breathalyzer alcohol concentration; OAGB, one-anastomosis gastric bypass; mo, months; *N* = 10.

**Table 3.** Breathalyzer alcohol PK parameters (SD) in each group

Parameter	Preoperative	1-mo postoperative	4-mo postoperative
AUC_0-inf_, g·min·L^−1^	21.0 (15.2)	71.2 (31.5)**	91.2 (39.3)**
C_max_, g·L^−1^	0.43 (0.33)	1.19 (0.30)***	1.24 (0.36)**
T_max_, min	15	15	15
t_1/2_, min	20.7 (15.0)	34.1 (13.9)	43.4 (16.8)**

Values in parentheses indicate standard deviation (SD).

PK, pharmacokinetics; AUC_0-inf_, area under the concentration-time curve from time zero to infinity; C_max_, maximal concentration; T_max_, time of C_max_; t_1/2_, elimination half-life; mo, months.

*p < 0.05; **p < 0.01; ***p < 0.001.

**Fig. 2.**
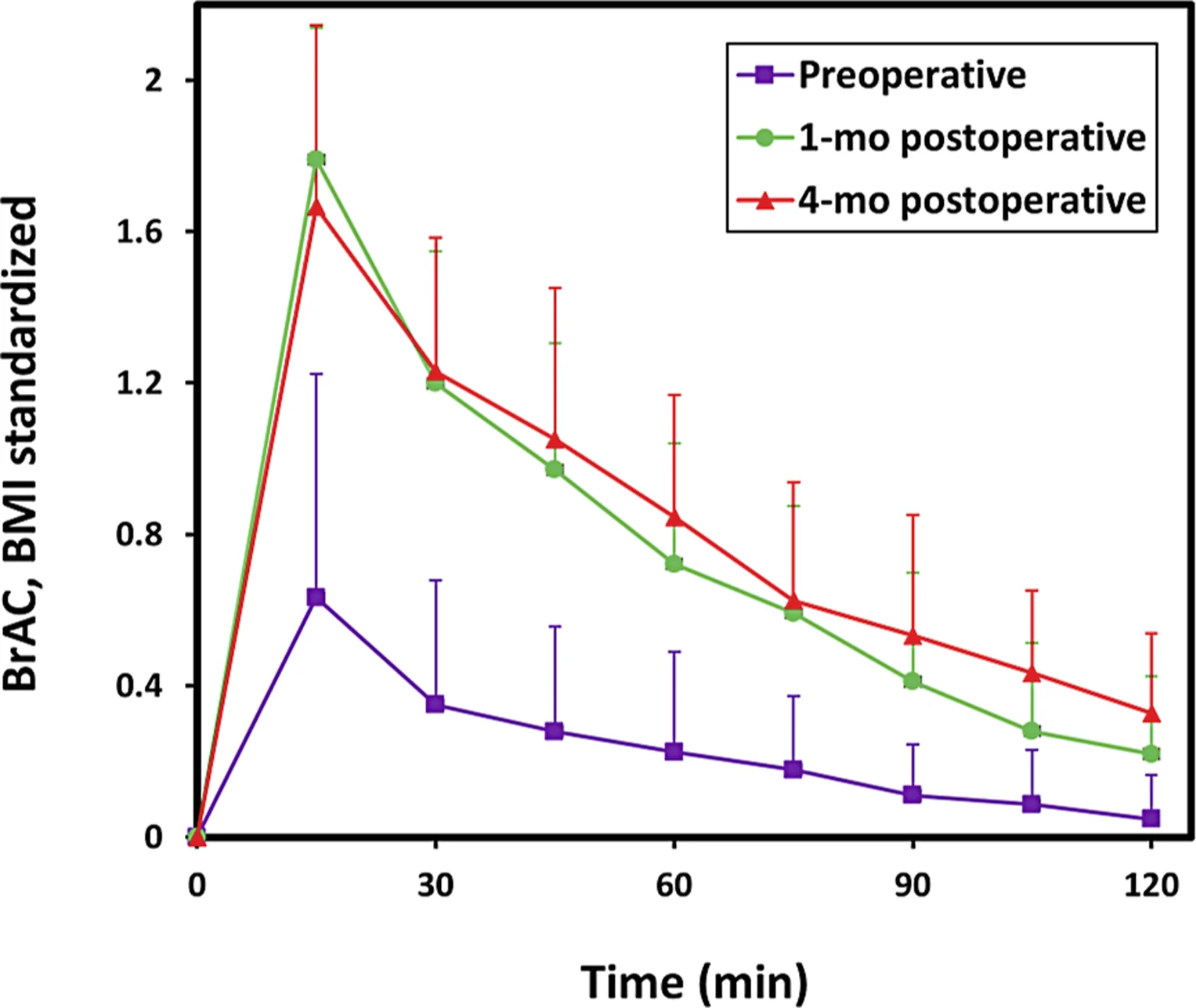
BMI-standardized BrAC-time profiles before surgery (purple squares) and at 1 month (green circles) and 4 months (red triangles) after OAGB, following wine ingestion. Bars represent standard deviation. BrAC, breathalyzer alcohol concentration; OAGB, one-anastomosis gastric bypass; mo, months; *N = 10*.

**Table 4.** BMI standardized breathalyzer alcohol PK parameters (SD) in each group

Parameter (adjusted)	Preoperative	1-mo postoperative	4-mo postoperative
AUC_0-inf_, g·min·L^−1^	32.1 (21.6)	105.6 (42.4)***	122.8 (55.9)**
C_max_, g·L^−1^	0.71 (0.53)	1.79 (0.33)**	1.67 (0.48)*
T_max_, min	15	15	15
t_1/2_, min	16.9 (10.9)	33.2 (14.1)	43.1 (17.2)**

Values in parentheses indicate standard deviation (SD).

PK, pharmacokinetics; AUC_0-inf_, area under the concentration-time curve from time zero to infinity; C_max_, maximal concentration; T_max_, time of C_max_; t_1/2_, elimination half-life; mo, months.

*p < 0.05; **p < 0.01; ***p < 0.001.

**Fig. 3.**
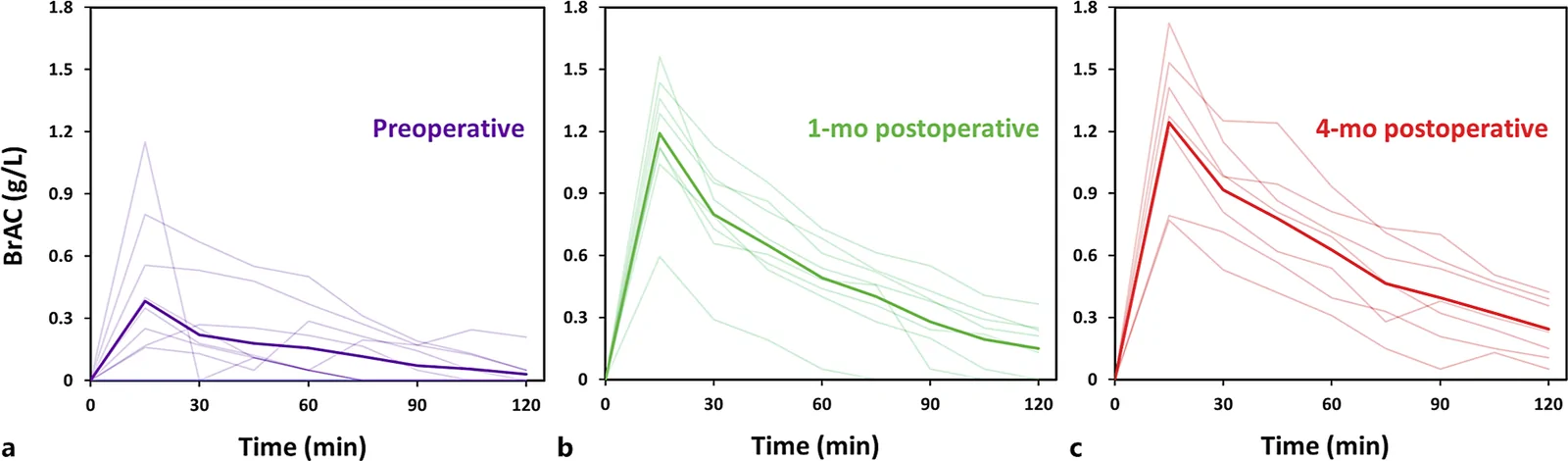
BrAC-time profiles of individual participants (transparent lines) before surgery *(N = 10)* (**a**) and at 1 month *(N = 8)* (**b**) and 4 months *(N = 7)* (**c**) after OAGB. The bold line represents the group mean profile. BrAC, breathalyzer alcohol concentration; OAGB, one-anastomosis gastric bypass; mo, months.

### Questionnaire Results

For the alcohol intoxication symptoms questionnaire, mean (SD) scores were 16.6 (3.7) at the preoperative assessment, 19.9 (3.7) at 1 month, and 24.0 (7.8) at 4 months postoperatively. An upward trend was observed from pre-OAGB to 1 month post-OAGB (mean difference: 95% CI *p* = 0.13), and a significant increase was noted from the preoperative assessment to 4 months postoperatively (*p* = 0.048). No significant difference was observed between the 1- and 4-month postoperative assessments (Fig. 4).

**Fig. 4.**
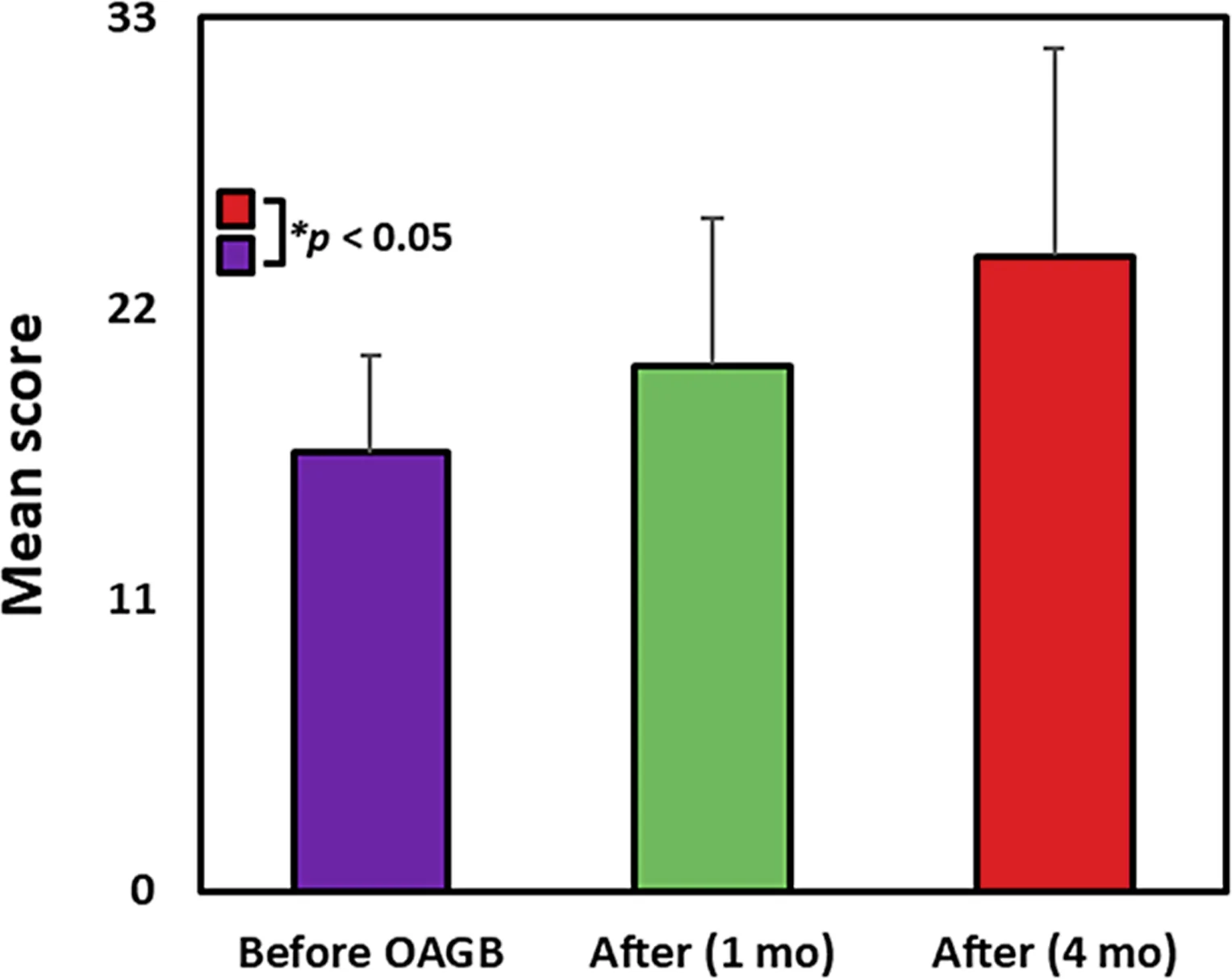
Mean questionnaire scores in participants before OAGB (*N* = 9) and at 1 month (*N* = 8) and 4 months (*N* = 7) after the procedure.

Figure 5 presents the PK-PD correlation, demonstrating a statistically significant positive association between the questionnaire score and each of the three PK parameters (C_max_, AUC_0-inf_, and C_60 min_). Among these, C_max_ was the strongest predictor of higher questionnaire scores (*R* = 0.422, *p* = 0.00007).

**Fig. 5.**
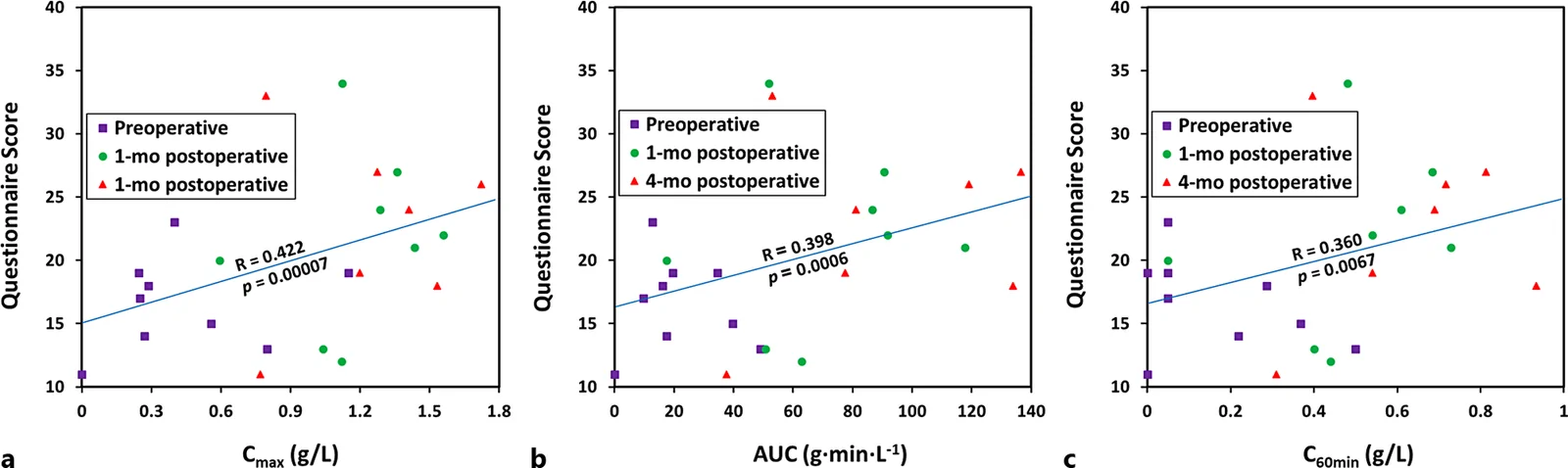
PK-PD correlations between the PK parameters C_max_ (**a**), AUC_0-inf_ (**b**), and C_60 min_ (**c**), and questionnaire scores before and at 1 month and 4 months after OAGB. Questionnaires were initiated 60 min after wine ingestion. The R value represents the strength of the correlation, while the *p* value indicates its statistical significance. PK, pharmacokinetics; PD, pharmacodynamics.

## Discussion

The PK of ethanol has been extensively studied in healthy individuals [32], as well as in patients after RYGB and SG. However, this exploratory study is the first to characterize ethanol PK following OAGB. Comparison of preoperative and postoperative data revealed marked differences in alcohol absorption and elimination. Specifically, higher C_max_ values were observed at both 1 month and 4 months after surgery, accompanied by a prolonged time to complete ethanol elimination.

Several mechanisms may account for these observations [33, 34]. First, the markedly reduced gastric volume after OAGB facilitates rapid emptying of fluids into the mid-jejunum [23, 35], thereby accelerating ethanol absorption. In practical terms, when alcohol is ingested, the small gastric pouch fills quickly and empties more rapidly into the small intestine. Rapid passage may also reduce first-pass metabolism (FPM). Ethanol FPM occurs primarily in the stomach [36] via gastric alcohol dehydrogenase (ADH), in addition to hepatic metabolism [22, 37]. Following OAGB, the marked reduction in gastric volume limits the availability and activity of gastric ADH, further contributing to decreased FPM [24, 38]. In addition, decreased alcohol clearance may be related to the loss of fat-free mass [17]. Other factors that may further enhance systemic ethanol exposure after MBS include substantial weight loss, altered hydration status, and reductions in muscle mass [19, 39].

In our exploratory study, C_max_ nearly tripled following OAGB, rising from 0.43 (0.33) g·L^−1^ preoperatively to 1.19 (0.30) g·L^−1^ at 1 month and 1.24 (0.36) g·L^−1^ at 4 months postoperatively. Additionally, the elimination half-life (t_1/2_) increased from 20.7 (15.0) min before surgery to 34.1 (13.9) and 43.4 (16.8) min after 1 month and 4 months, respectively. These results align with previous reports demonstrating elevated C_max_ values and delayed ethanol elimination several months after gastric bypass surgery. Woodard et al. [13] reported significantly higher peak BrACs at 3 months (0.59 g·L^−1^) and 6 months (0.88 g·L^−1^) after RYGB compared with presurgery levels (0.24 g·L^−1^). Molina-Castro et al. [17] reported maximal BAC of 0.68 g·L^−1^ before MBS and 1.07 g·L^−1^ around 5 months after MBS (either RYGB or SG), with ∼28% decrease in alcohol clearance. Overall, alterations in ethanol PK following MBS appear most pronounced in the early postoperative period and may attenuate over time. Furthermore, compared with SG, RYGB has been associated with larger postoperative increases in ethanol C_max_ relative to preoperative values (Table 1).

In a descriptive sex-stratified analysis, increased postoperative BrAC levels were observed in both males and females (data not shown). Females tended to have higher BrAC levels across study visits; however, the small sample size did not allow formal assessment of sex-related differences.

In addition to the PK alterations, changes were also observed in patient-reported alcohol intoxication symptoms. The mean questionnaire scores increased from 16.6 preoperatively to 24.0 (*p* < 0.05) at 4 months postoperatively. These findings suggest that the increased systemic exposure to ethanol following OAGB may be clinically reflected in stronger subjective intoxication symptoms.

The clinical implications of these findings are particularly relevant for patients undergoing OAGB. In the present study, consumption of the same amount of alcohol before and after surgery resulted in substantially higher postoperative BrAC levels, greater systemic ethanol exposure, and more pronounced subjective alcohol effects. These findings indicate that alcohol responses following OAGB may differ considerably from those experienced before surgery.

From a practical standpoint, patients should be informed that alcohol handling may change significantly after OAGB. The marked increase in postoperative BrAC levels observed in this study suggests that a given amount of alcohol may result in substantially higher alcohol exposure than before surgery. Such changes may have implications for activities requiring unimpaired psychomotor performance, including driving. Accordingly, alcohol-related counseling should be considered as part of routine preoperative and postoperative patient education [40, 41].

The present findings may also be relevant to observations reported after other bariatric procedures, particularly bypass operations, where increased rates of alcohol-related complications and AUD have been described. Although the current study did not evaluate alcohol consumption patterns, AUD, or other behavioral outcomes, the marked increase in ethanol exposure after OAGB may represent one potential biological mechanism contributing to these observations. Further studies are required to determine whether the PK changes observed after OAGB translate into clinically meaningful long-term outcomes.

Additional long-term considerations warrant attention. Observational studies have reported associations between bariatric surgery, alcohol-related liver disease, and other alcohol-related complications [42]. Furthermore, alcohol provides a significant caloric load, and regular alcohol consumption has been proposed as one of several factors that may contribute to suboptimal weight-loss outcomes or weight regain after bariatric surgery [43]. The present study was not designed to assess hepatic outcomes, weight trajectories, or long-term alcohol-related complications. Therefore, these issues should be viewed as potential considerations rather than direct consequences demonstrated by the current findings. Nevertheless, the substantial increase in ethanol exposure observed after OAGB highlights the importance of addressing alcohol consumption during long-term postoperative follow-up.

## Limitations

This exploratory study has several limitations. First, the sample size was small (*n* = 10), with participant attrition during follow-up, which limits the generalizability of the findings. Second, methodological differences in T_max_ assessment should be considered [22]. Unlike blood-based studies, which can obtain samples immediately after alcohol ingestion, breathalyzer measurements require a delay following drinking to avoid contamination from residual alcohol in the oral cavity. As a result, the true peak alcohol concentration may have occurred earlier than the first post-ingestion measurement, potentially contributing to the longer T_max_ observed in our study (15 min vs. 5–10 min in blood-based studies [14, 23]). Nevertheless, our findings remained statistically significant, and it is reasonable to assume that blood-based measurements would have yielded even higher C_max_ values. Also, the present study used a standardized 5-min alcohol administration protocol to minimize variability between visits and participants. In reality, people often take longer to drink wine or other alcoholic beverages. Whether slower alcohol consumption would attenuate the observed postoperative increase in BrAC remains a question for future investigation. Additionally, the absence of a control group limits causal inference. And finally, the postoperative observation period (up to 4 months postoperatively) was relatively short, so longer-term alterations in alcohol PK after OAGB remain to be determined.

## Conclusions

In conclusion, alcohol PK is altered following the OAGB procedure. Patients should be aware that even modest alcohol consumption (e.g., 200 mL of 13.5% ethanol) may potentially lead to unexpectedly high BrAC levels. These findings reflect the importance of clinician-led patient education and advice on safer alcohol use after surgery.

## Statement of Ethics

The study was approved by the Institutional Review Board (Helsinki Committee) of Soroka University Medical Center, Approval #SOR-20-0324. Written informed consent was obtained from all participants.

## Conflict of Interest Statement

All authors declare no conflicts of interest related to this study.

## Funding Sources

This research received no external funding.

## Author Contributions

E.Q., D.P., A.D., and O.D. conceived and designed the study. E.Q., D.P., I.P., and A.W. performed statistical analysis. E.Q., D.P., A.E.D., I.K., A.D., and O.D. performed acquisition, analysis, and interpretation of data and wrote the paper. E.Q., D.P., A.E.D., I.P., A.W., I.K., A.D., and O.D. reviewed the manuscript for important intellectual content, approved the final version for publication, and agreed to be accountable for all aspects of the work in ensuring that questions related to the accuracy or integrity of any part of the work are appropriately investigated and resolved.

## Data Availability

All data generated or analyzed during this study are included in this article. Further inquiries can be directed to the corresponding author.
